# Supplementary material for: Charge guides pathway selection in β-sheet fibrillizing peptide co-assembly
Source: Commun Chem. 2020 Nov 13;3:172. doi: 10.1038/s42004-020-00414-w (PMC9814569; doi:10.1038/s42004-020-00414-w)
Supplement: Supplementary file 1 — Supplementary Information [file 42004_2020_414_MOESM1_ESM.pdf]

Supplementary Information for

Charge guides pathway selection in β-sheet fibrillizing peptide co-assembly

Authors: Dillon T. Seroski[1], Xin Dong[2], Kong M. Wong[3], Renjie Liu[1], Qing Shao[2], Anant K. Paravastu[3], Carol K. Hall[2], Gregory A. Hudalla[1*]

Affiliations:

1. J. Crayton Pruitt Family Department of Biomedical Engineering, University of Florida, Gainesville, Florida 32611, United States

2. Department of Chemical and Biomolecular Engineering, North Carolina State University, 911 Partners Way, Raleigh, North Carolina 27695, United States

3. School of Chemical and Biomolecular Engineering, Georgia Institute of Technology, Atlanta, Georgia, 30332, United States

Keywords: Peptide, co-assembly, charge-complementarity, kinetics, self-assembly

***Corresponding Author**:

Gregory A. Hudalla, PhD

Biomedical Sciences J293, PO BOX 116131

1275 Center Drive

Gainesville, FL 32611

ghudalla@bme.ufl.edu

(352) 273-9326

Supplementary Figures

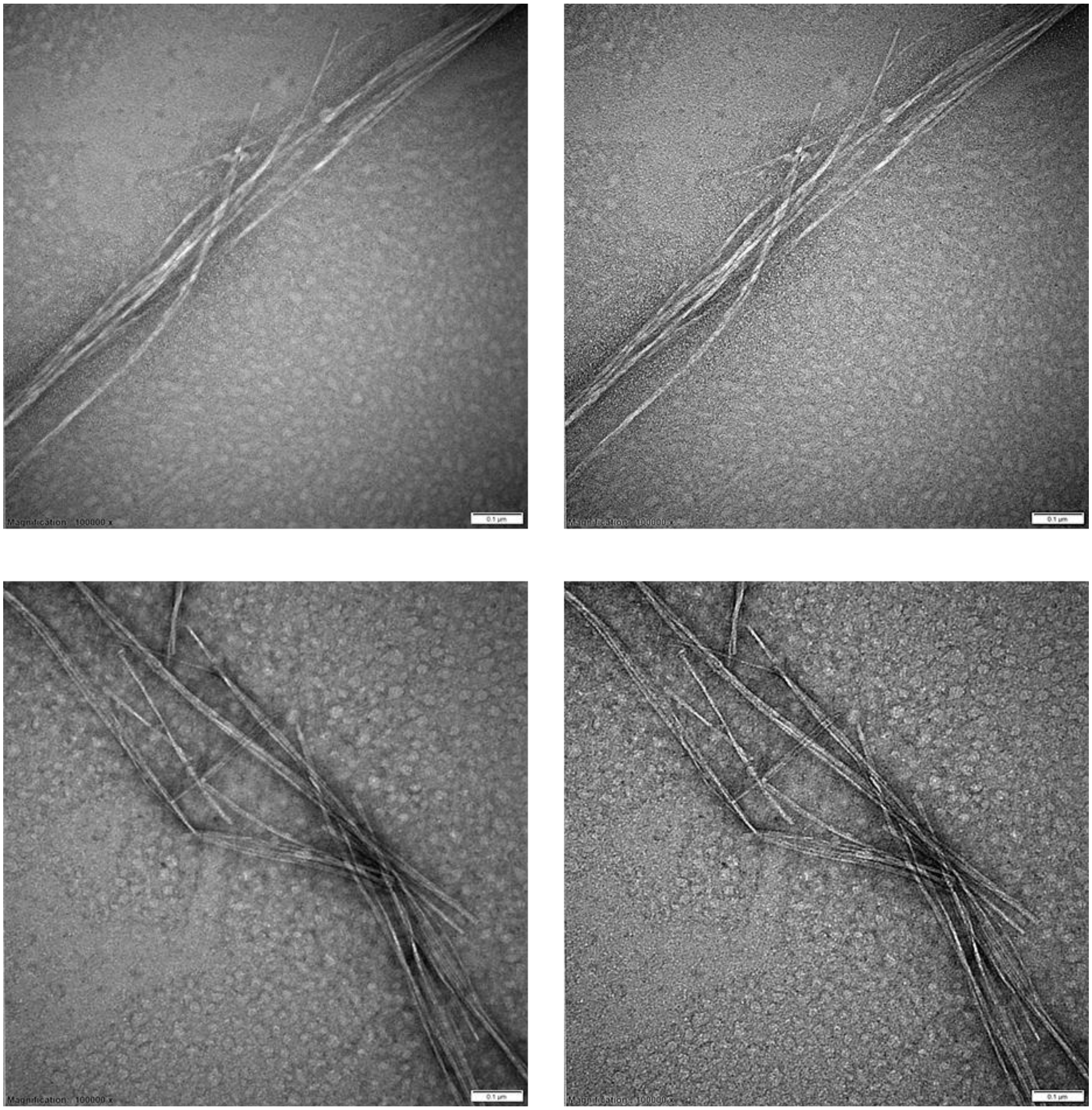

Supplementary Fig. 1: CATCH(2+/2-) TEM micrograph unmodified (left) and 50% sharpened (right) using Microsoft PowerPoint.

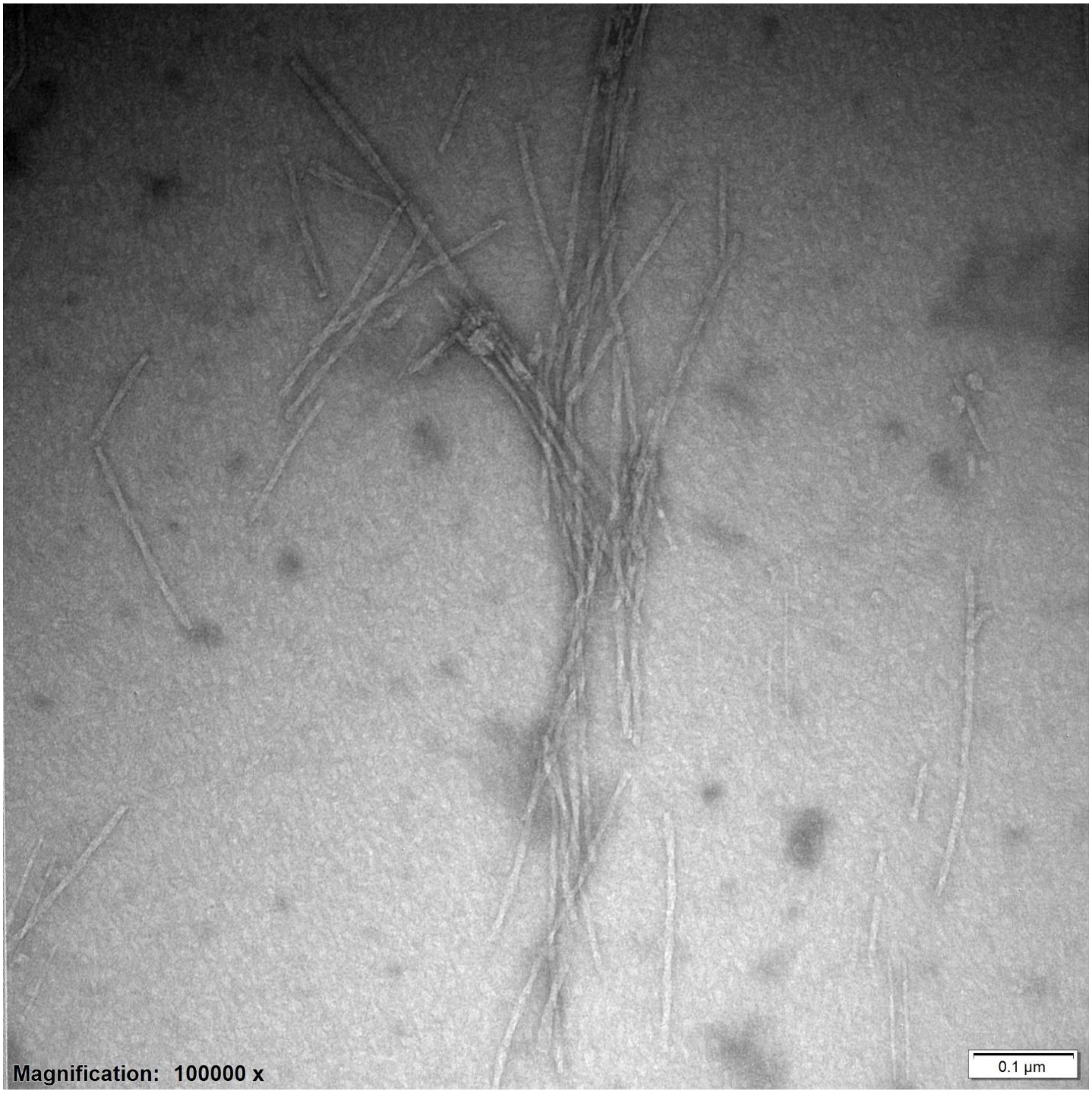

Supplementary Fig. 2: TEM micrograph of Q11 nanofibers.

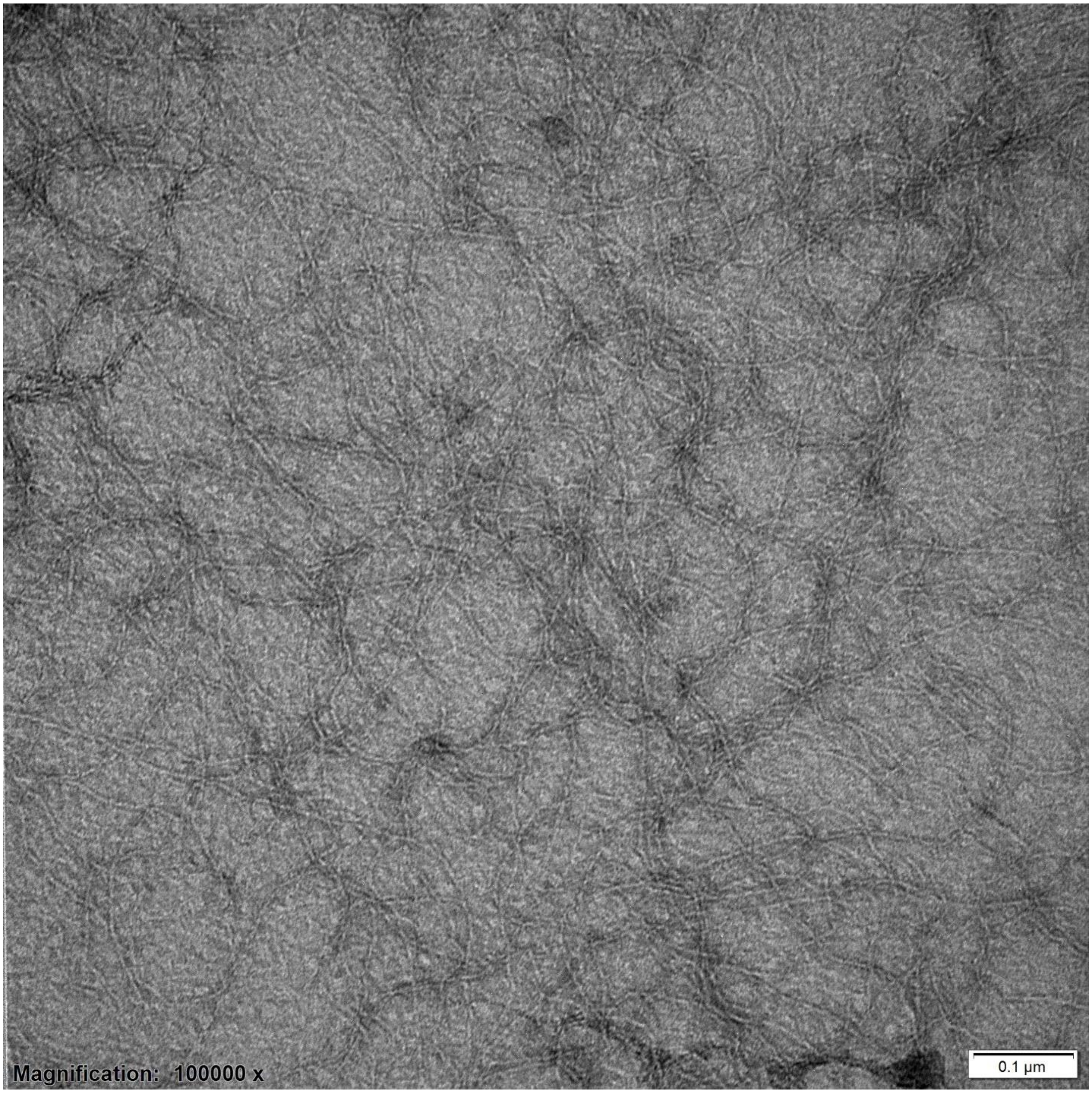

Supplementary Fig. 3: CATCH(6+/6-) TEM micrograph unmodified.

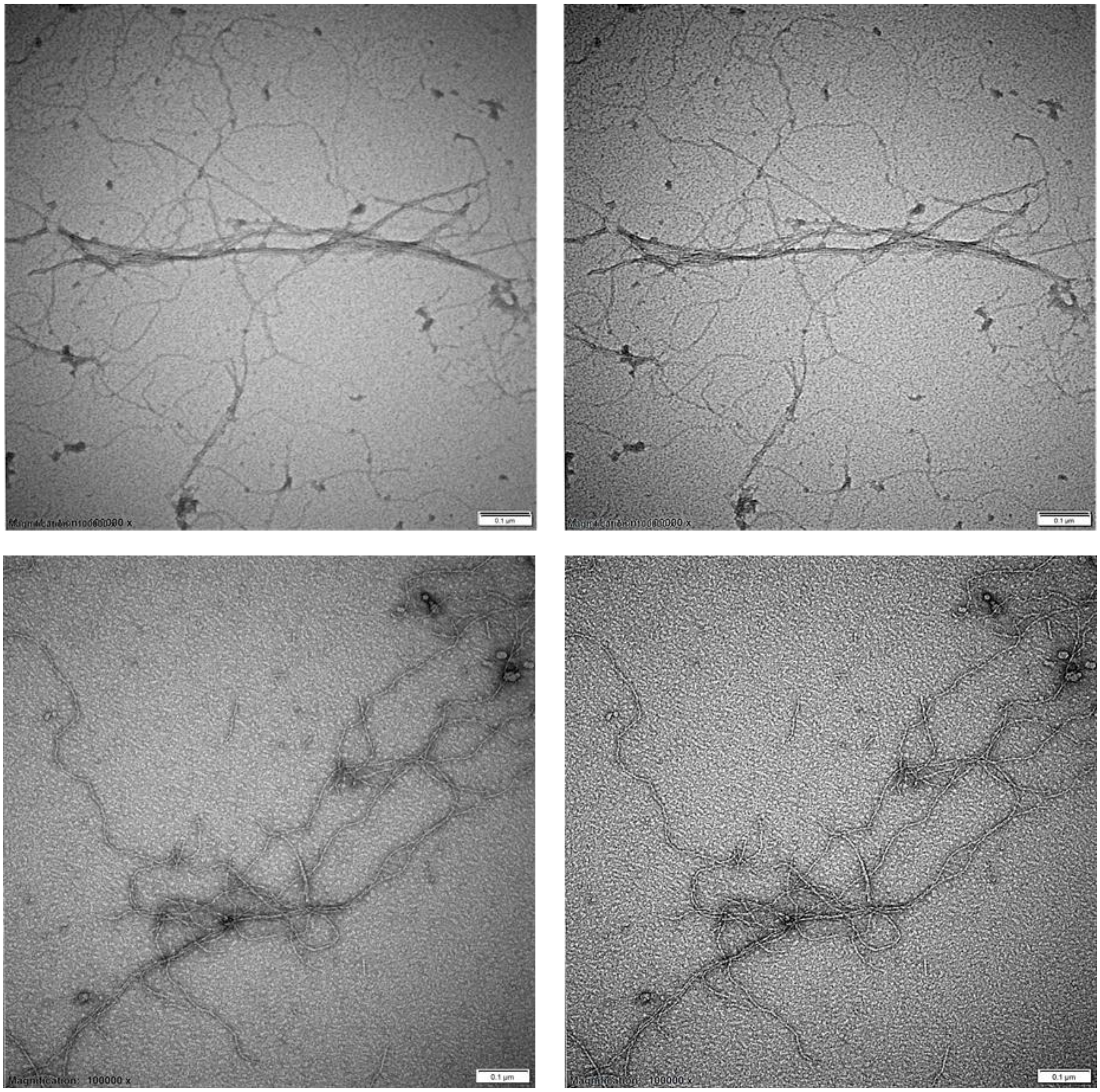

Supplementary Fig. 4: CATCH(4+/4-) TEM micrograph unmodified (left) and 50% sharpened (right) using Microsoft PowerPoint.

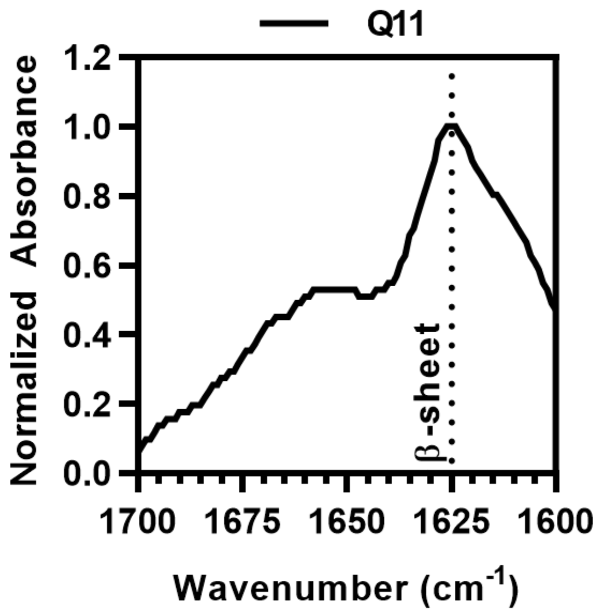

Supplementary Fig. 5: FTIR spectra of 10 mM Q11.

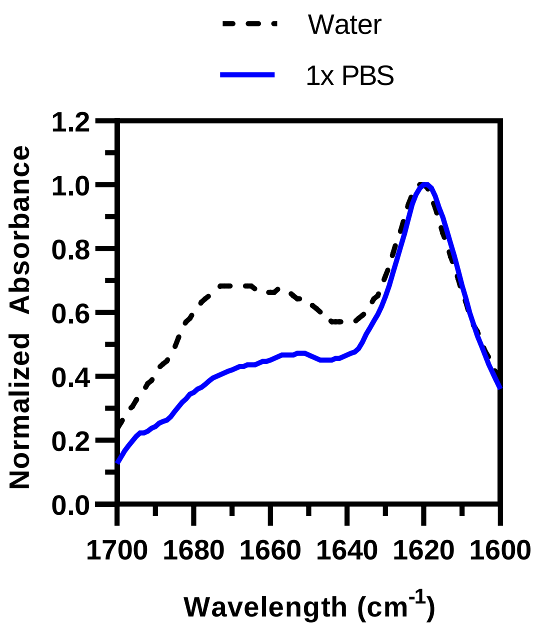

Supplementary Fig. 6: Normalized FTIR spectra of 10 mM CATCH(2+) in water (dashed black line) or 1x PBS (solid blue line).

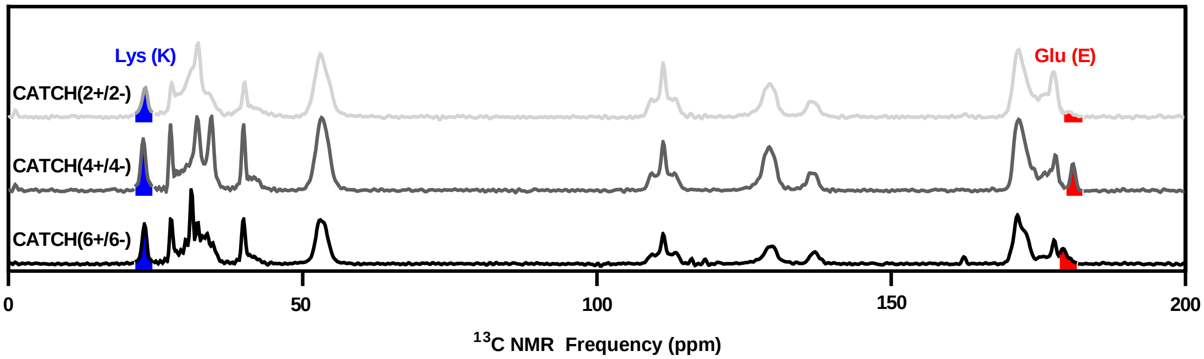

Supplementary Fig. 7: Full 1D NMR spectra of CATCH(2+/2-) (light gray line), CATCH(4+/4-) (dark gray line), and CATCH(6+/6-) (black line).

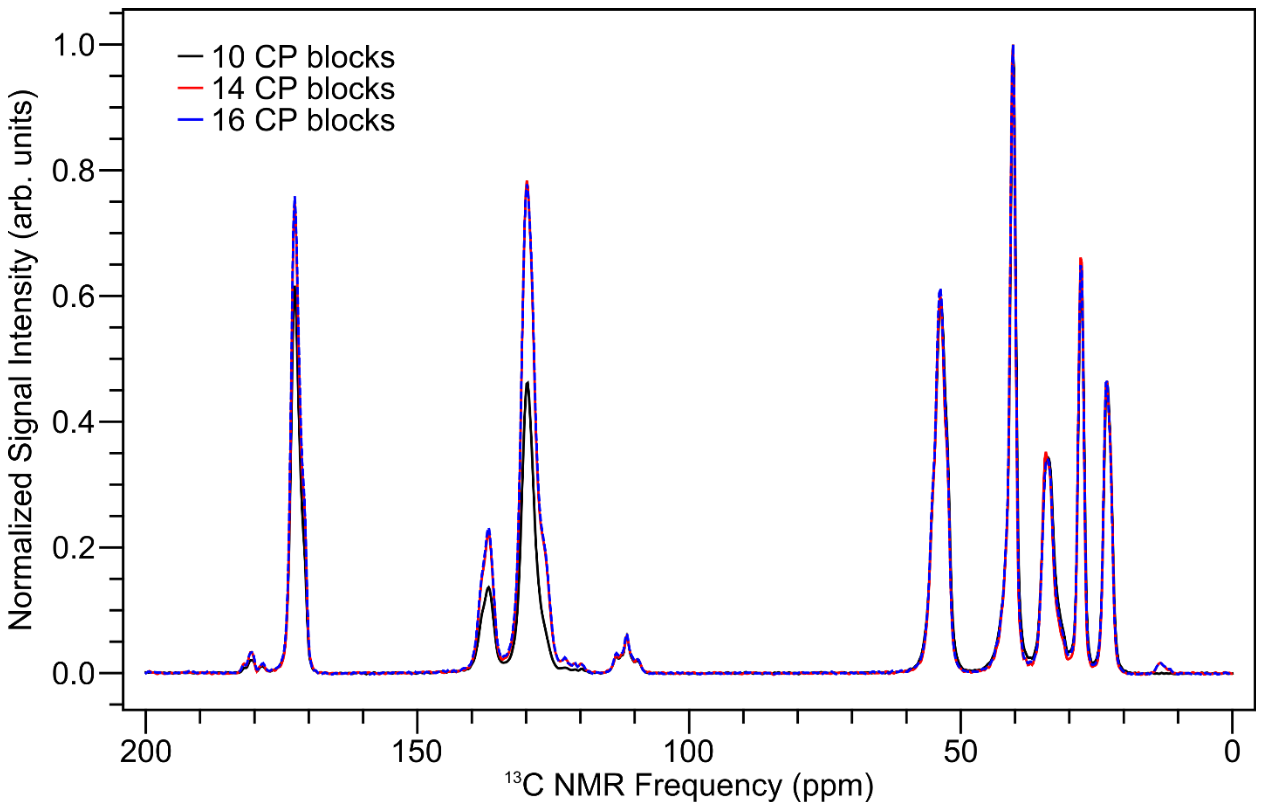

Supplementary Fig. 8: Effect of the number of CP blocks on ^{13}C multiCP spectra of a coassembled King-Webb peptide nanofiber uniformly labelled with ^{13}C and ^{15}N at F3 and K9. 10 CP blocks, gray dashed line; 14 CP blocks, red dashed line; 16 CP blocks, blue dashed line.

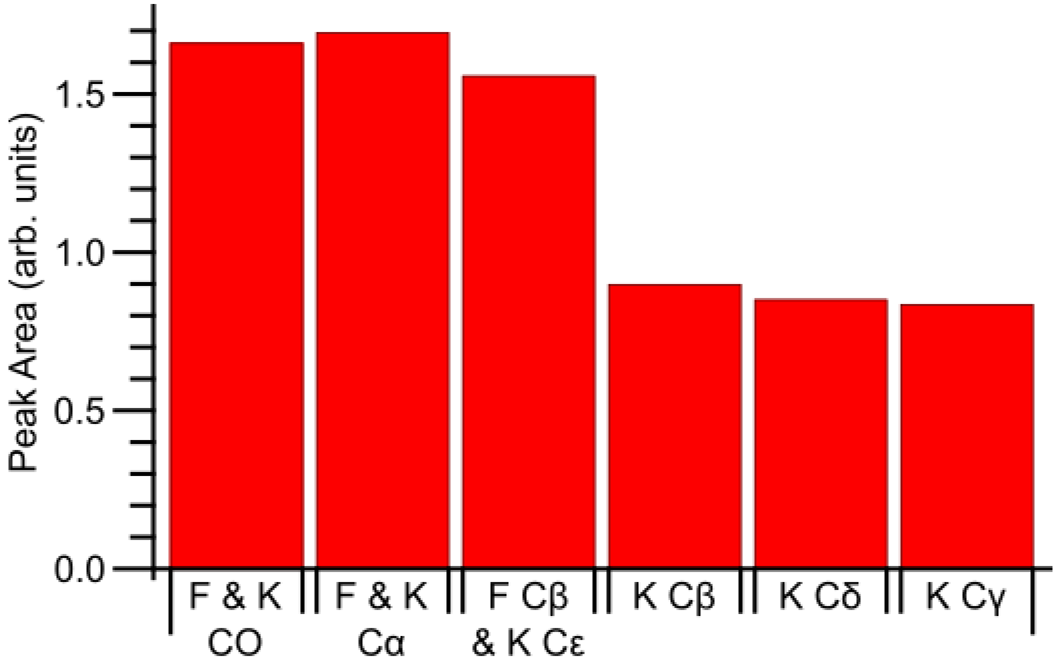

Supplementary Fig. 9: Fitted peak areas from the 14 CP block run in Figure S1. Peaks were fitted to gaussian distributions.

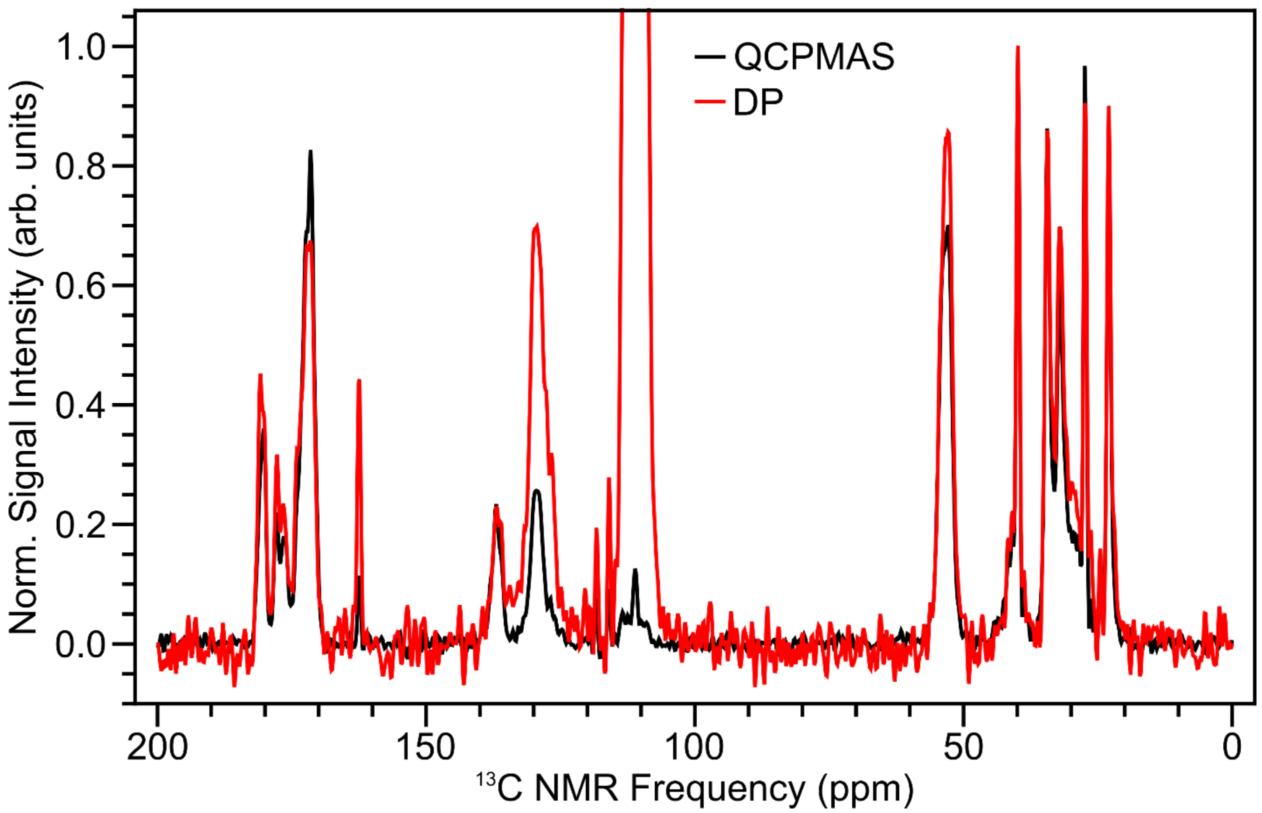

Supplementary Fig. 10: Comparison of 1D ^{13}C NMR spectra of a co-assembled β-sheet peptide nanofiber produced from an equimolar CATCH6K and CATCH6E mixture. The black trace corresponds to measurements conducted using the composite pulse multiCP pulse sequence. The red trace corresponds to measurements performed with a direct pulse and 42 s recycle delay. QCPMAS, black line; DP, red line.

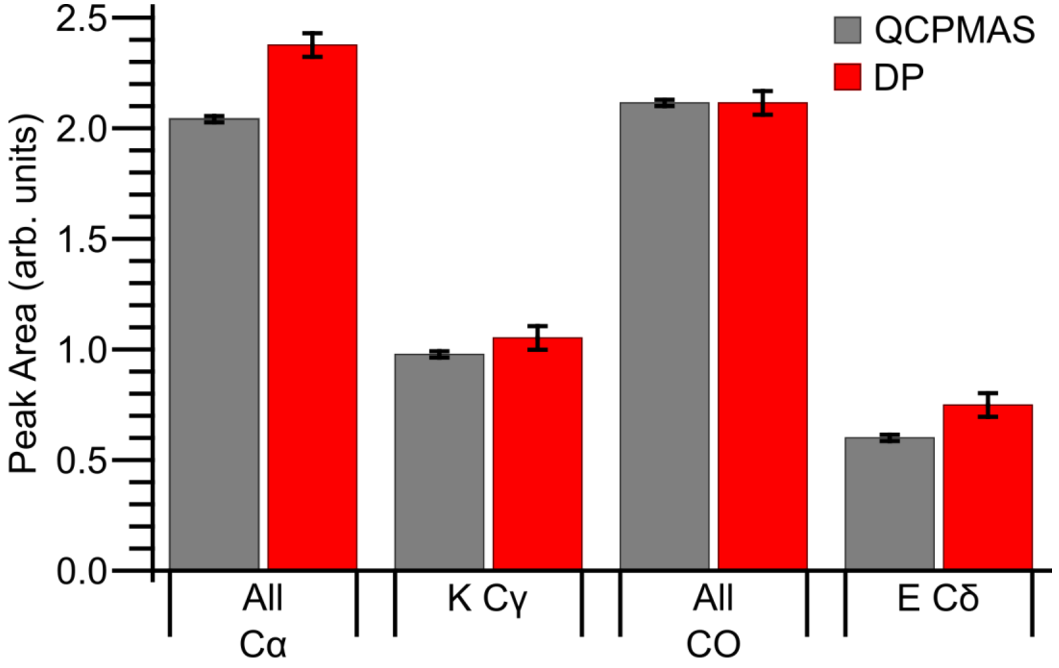

Supplementary Fig. 11: Comparison of fitted peak areas measured from the 1D ^{13}C NMR spectra using QCPMAS or DP methods shown in Figure S3. Peaks were fitted to gaussian distributions and error bars show 95% confidence intervals determined from background noise. QCPMAS, gray bars; DP, red bars.

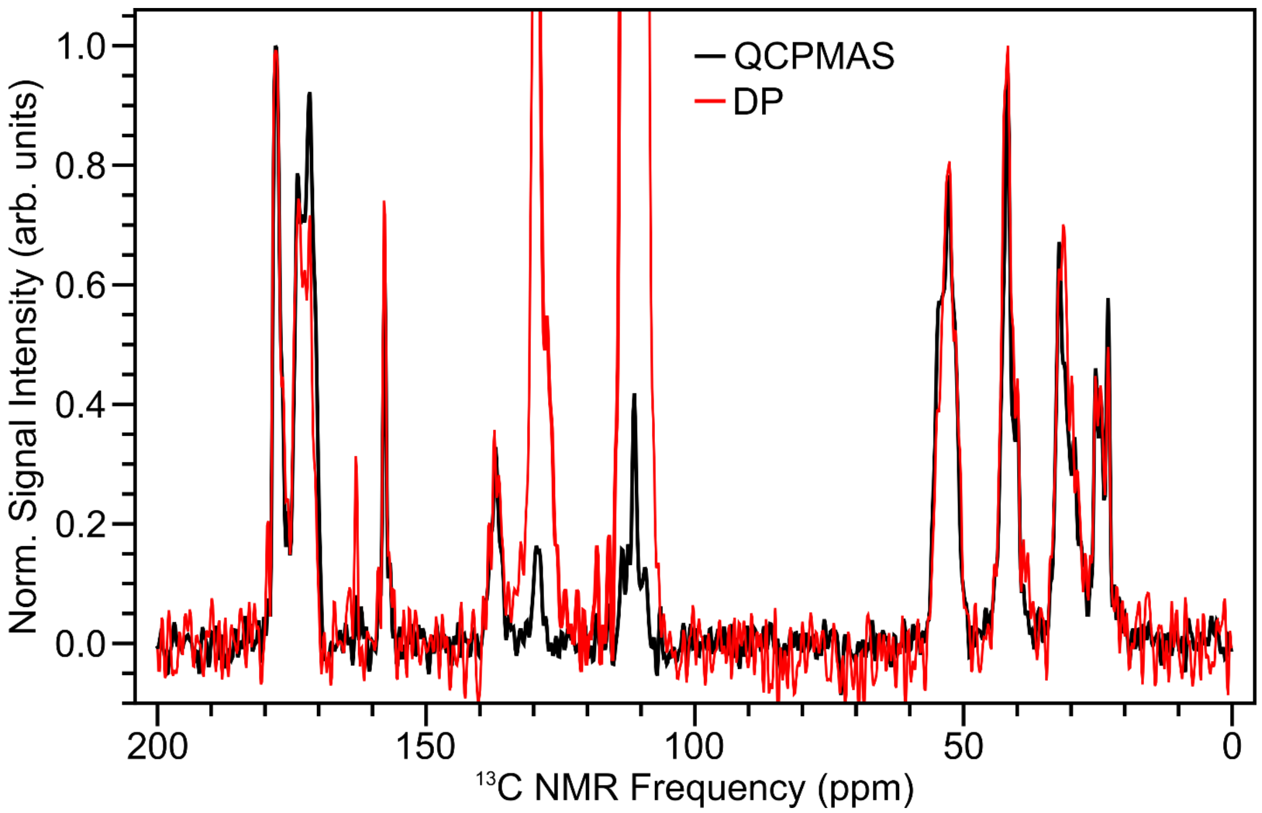

Supplementary Fig. 12: Comparison of 1D ^{13}C NMR spectra of a co-assembled β-sheet peptide nanofiber produced from an equimolar CATCH6R and CATCH6D mixture. The black trace corresponds to measurements conducted using the composite pule multiCP pulse sequence. The red trace corresponds to measurements performed with a direct pulse and 42 s recycle delay. QCPMAS, black line; DP, red line.

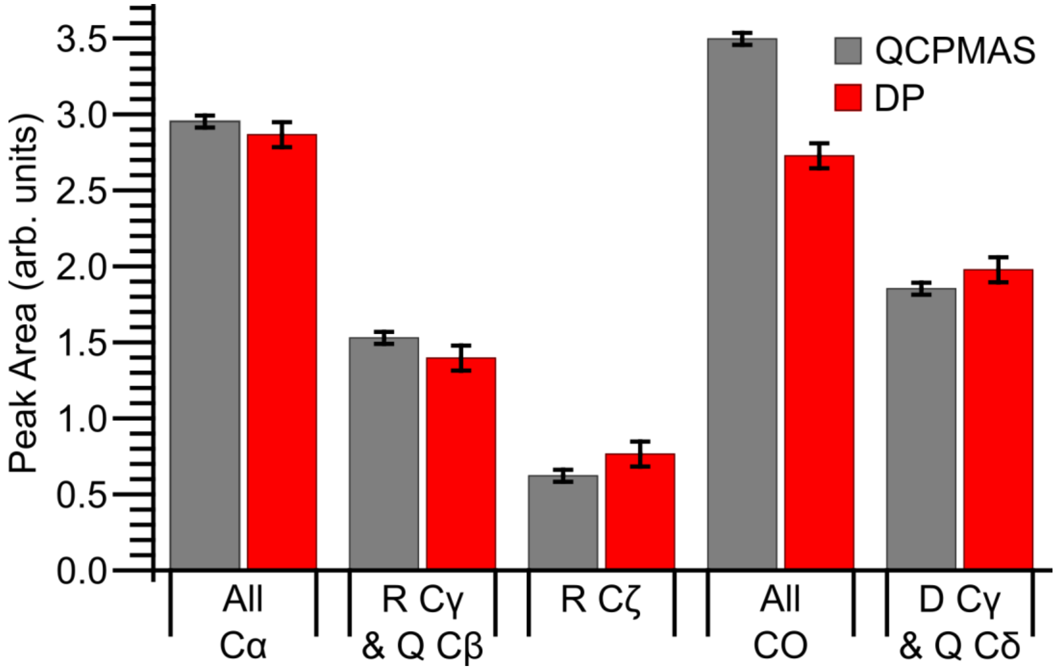

Supplementary Fig. 13: Comparison of fitted peak areas measured from the 1D ^{13}C NMR spectra using QCPMAS or DP methods shown in Figure S5. Peaks were fitted to gaussian distributions and error bars show 95% confidence intervals determined from background noise. QCPMAS, gray bars; DP, red bars.

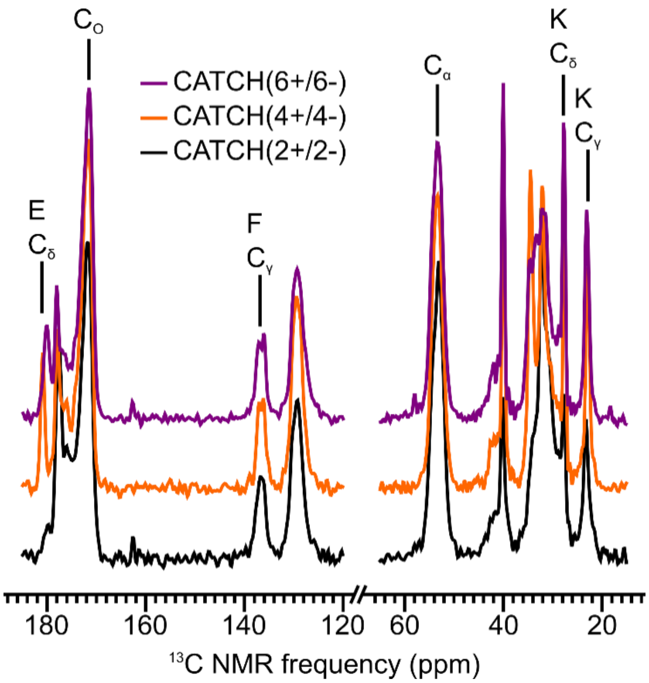

Supplementary Fig. 14: Overlay of 1D ^{13}C NMR spectra of CATCH(2+/2-) (black line), CATCH(4+/4-) (orange line), and CATCH(6+/6-) (purple line). Peaks analyzed in Supp. Table 1 are labeled.

CATCH(2+/2-), t = 16 μs

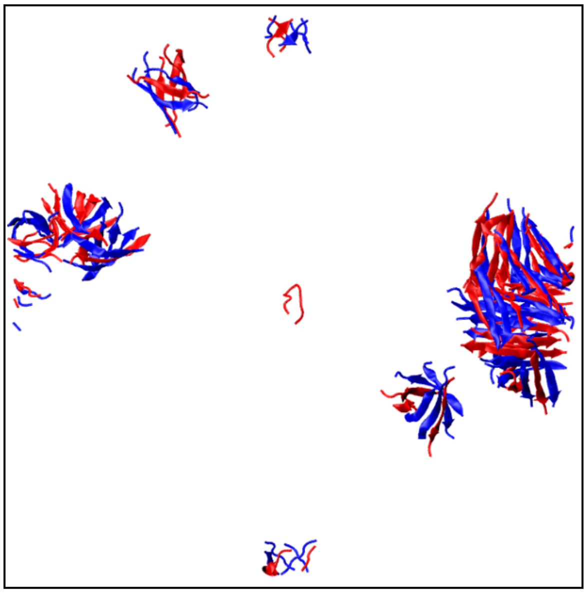

Supplementary Fig. 15: DMD simulations of CATCH(2+/2-) at T = 0.18. CATCH(2+) shown as blue and CATCH(2-) shown as red.

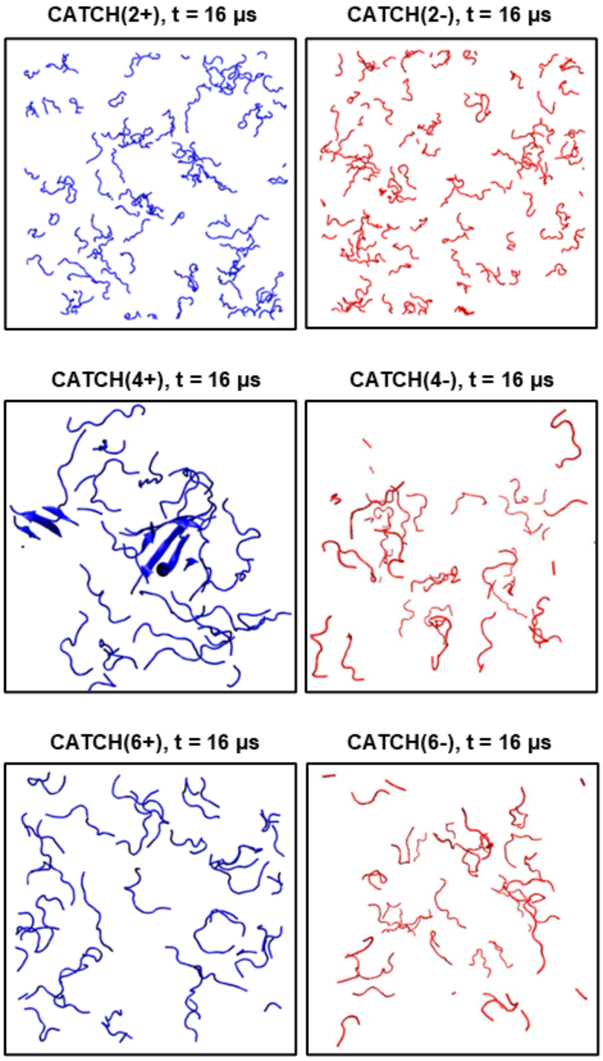

Supplementary Fig. 16: DMD simulations of CATCH peptides alone at T = 0.2.

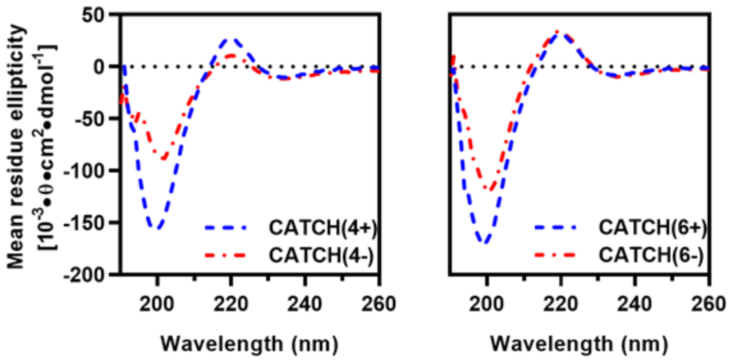

Supplementary Fig. 17: CD spectra of CATCH peptides alone. CATCH(4+) and CATCH(6+) shown as blue dashed lines. CATCH(4-) and CATCH(6-) shown as red dashed lines.

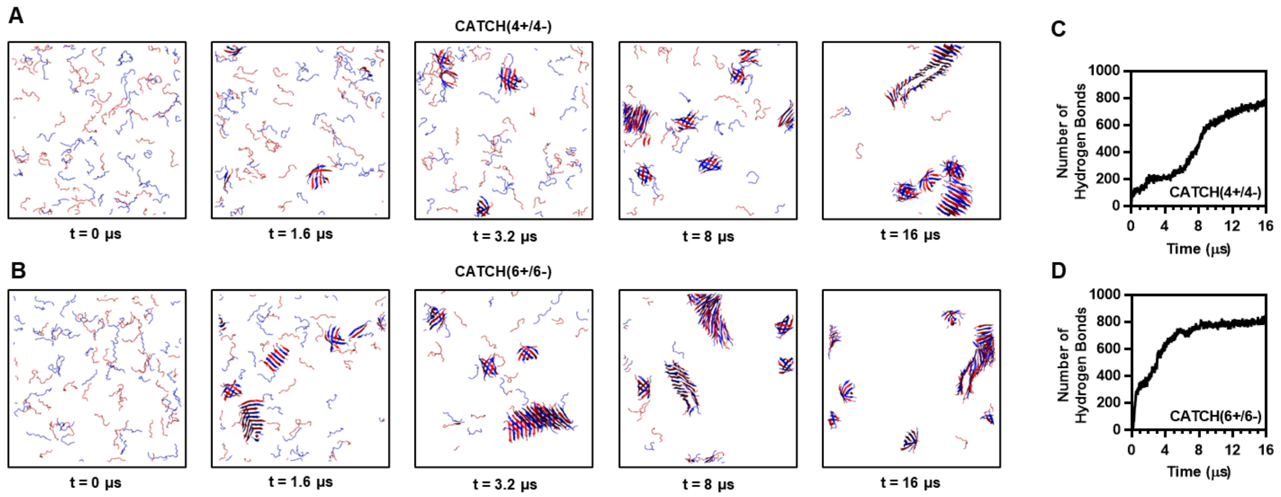

Supplementary Fig. 18: Replicate of DMD simulations. DMD snapshots of (A) CATCH(4+/4-) and (B) CATCH(6+/6-). Number of hydrogen bonds over time from DMD simulations for (C) CATCH(4+/4-) and (D) CATCH(6+/6-).

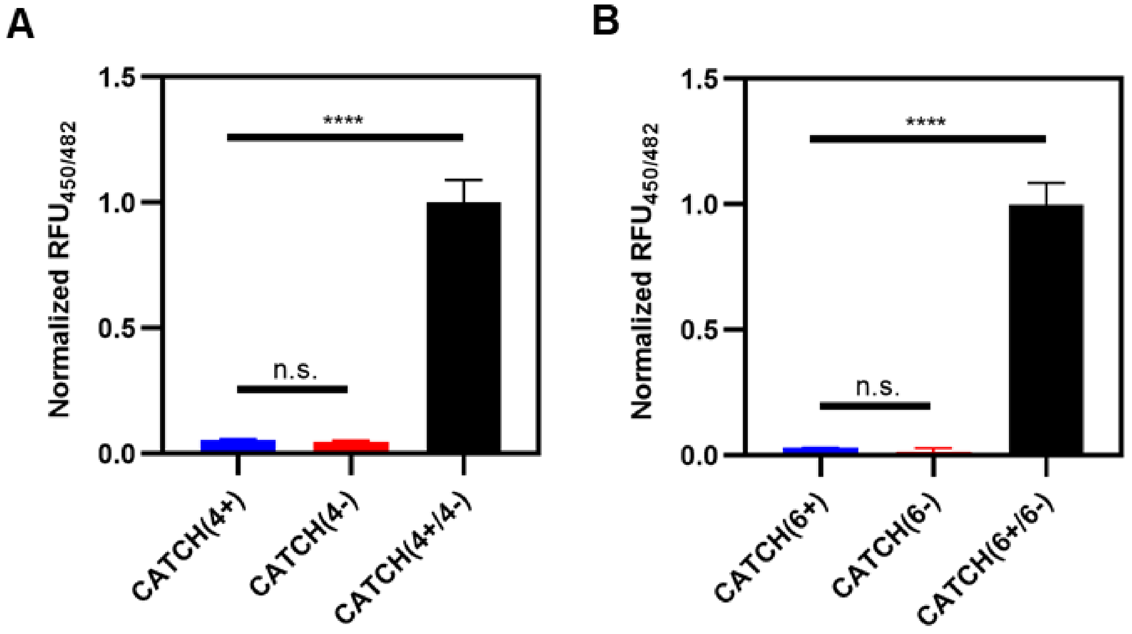

Supplementary Fig. 19: Thioflavin T endpoint assay of (A) CATCH(4+) (blue bar), CATCH(4-) (red bar), and CATCH(4+/4-) (black bar) and (B) CATCH(6+) (blue bar), CATCH(6-) (red bar), and CATCH(6+/6-) (black bar). ****$p<0.0001$ using Tukey's multiple comparisons test. Error bars shown as the standard error of the mean.

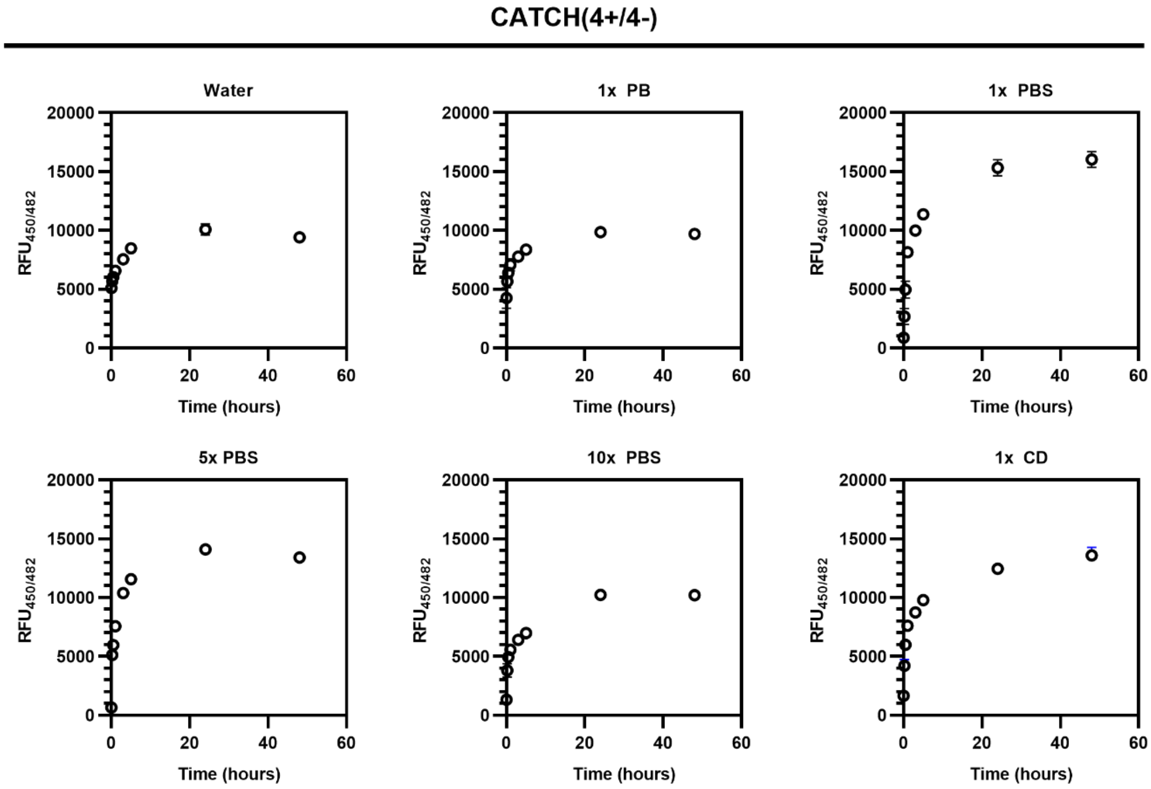

Time to half-max (hr)

Water	2.69±0.34
1x PB	2.01±1.09
1x PBS	2.14±0.06
5x PBS	1.11±0.54
10x PBS	3.21±0.79
1x CD	2.13±0.45

Supplementary Fig. 20: ThT kinetic profiles of CATCH(4+/4-) in water, 1x phosphate buffer (PB) (10 mM Na_2HPO_4 and 1.8 mM KH_2PO_4, pH 7.4), 1x PBS (137 mM NaCl, 2.7 mM KCL, 10 mM Na_2HPO_4 and 1.8 mM KH_2PO_4, pH 7.4), 5x PBS, 10x PBS, and 1x CD buffer (137 mM KF, 2.7 mM KCL, 10 mM Na_2HPO_4 and 1.8 mM KH_2PO_4, pH 7.4). Error bars shown as the standard error of the mean.

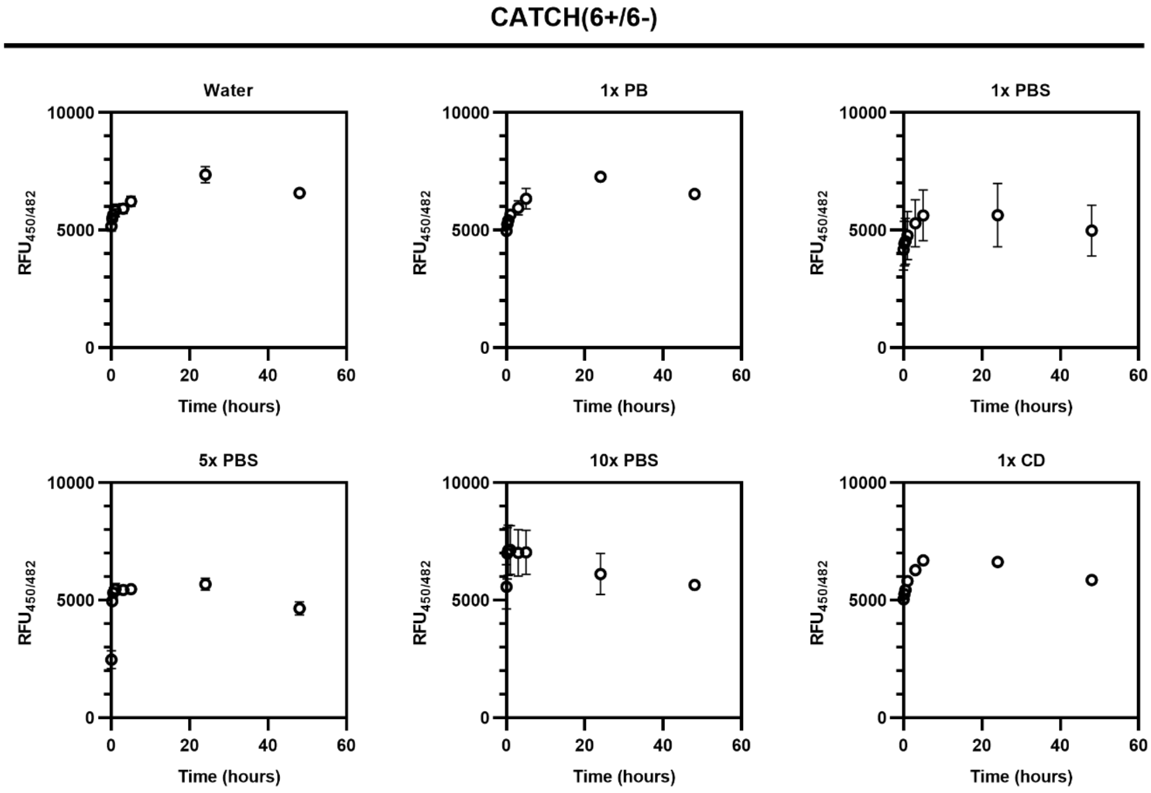

Time to half-max (hr)

Water	3.98±0.76
1x PB	3.08±1.69
1x PBS	0.91±0.91
5x PBS	0.08±0.007
10x PBS	Cannot be determined
1x CD	0.78±0.04

Supplementary Fig. 21: ThT kinetic profiles of CATCH(6+/6-) in water, 1x phosphate buffer (PB) (10 mM Na_2HPO_4 and 1.8 mM KH_2PO_4, pH 7.4), 1x PBS (137 mM NaCl, 2.7 mM KCL, 10 mM Na_2HPO_4 and 1.8 mM KH_2PO_4, pH 7.4), 5x PBS, 10x PBS, and 1x CD buffer (137 mM KF, 2.7 mM KCL, 10 mM Na_2HPO_4 and 1.8 mM KH_2PO_4, pH 7.4). *Fit not possible for 10x PBS due to rapid kinetics. Error bars shown as the standard error of the mean.

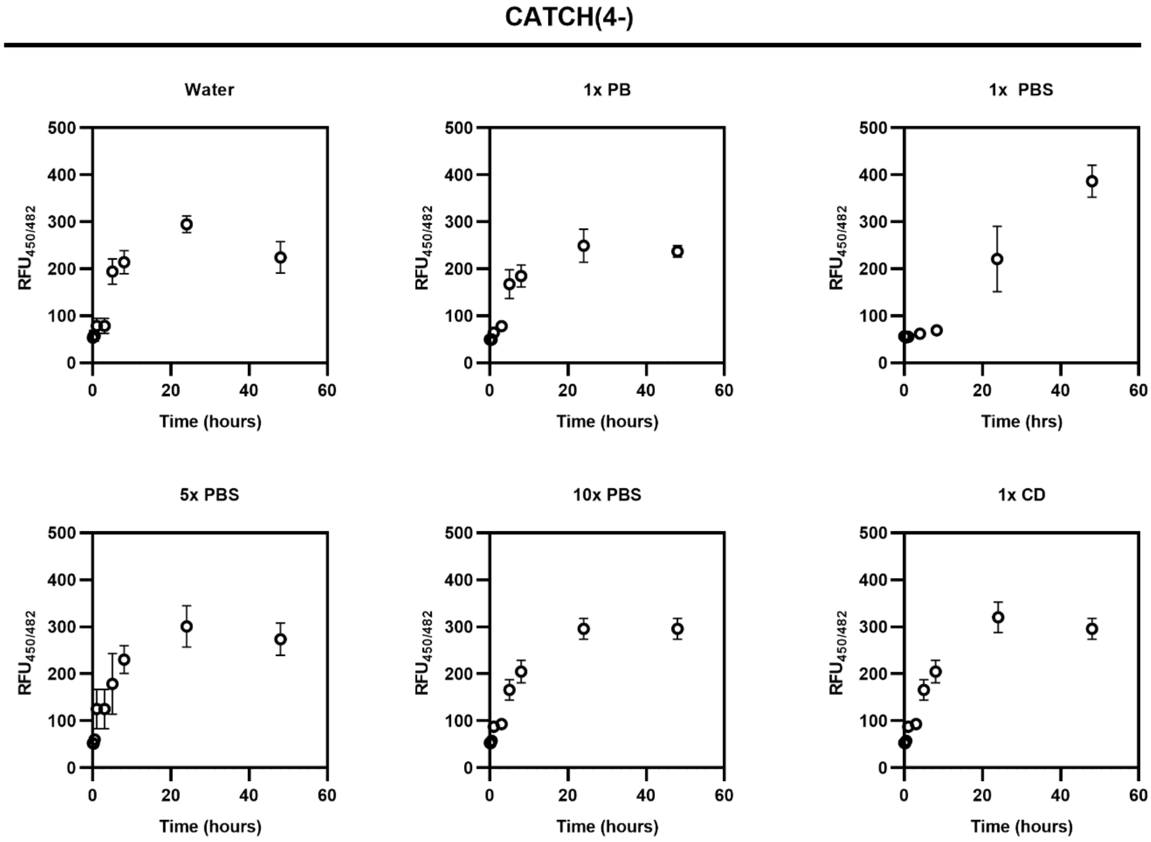

Supplementary Fig. 22: ThT kinetic profiles of CATCH(4-) in water, 1x phosphate buffer (PB) (10 mM Na_2HPO_4 and 1.8 mM KH_2PO_4, pH 7.4), 1x PBS (137 mM NaCl, 2.7 mM KCL, 10 mM Na_2HPO_4 and 1.8 mM KH_2PO_4, pH 7.4), 5x PBS, 10x PBS, and 1x CD buffer (137 mM KF, 2.7 mM KCL, 10 mM Na_2HPO_4 and 1.8 mM KH_2PO_4, pH 7.4). Error bars shown as the standard error of the mean.

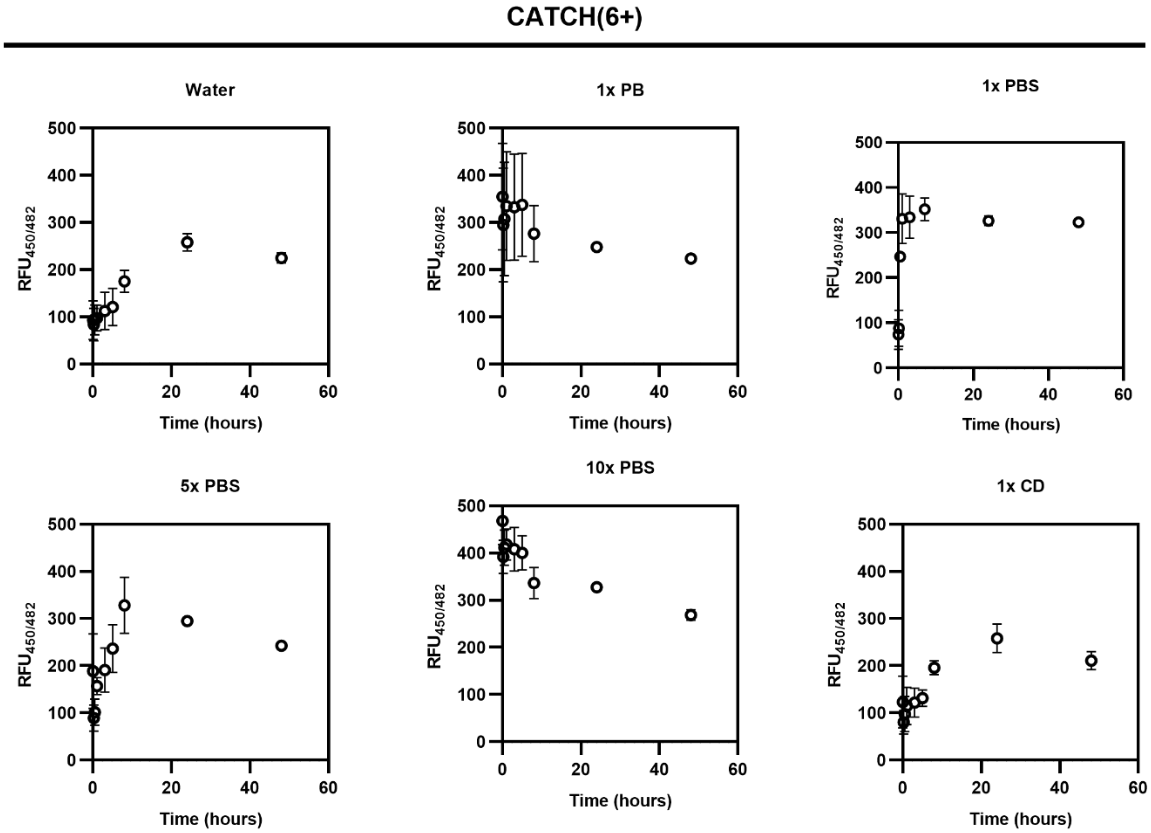

Supplementary Fig. 23: ThT kinetic profiles of CATCH(6+) in water, 1x phosphate buffer (PB) (10 mM Na_2HPO_4 and 1.8 mM KH_2PO_4, pH 7.4), 1x PBS (137 mM NaCl, 2.7 mM KCL, 10 mM Na_2HPO_4 and 1.8 mM KH_2PO_4, pH 7.4), 5x PBS, 10x PBS, and 1x CD buffer (137 mM KF, 2.7 mM KCL, 10 mM Na_2HPO_4 and 1.8 mM KH_2PO_4, pH 7.4). Error bars shown as the standard error of the mean.

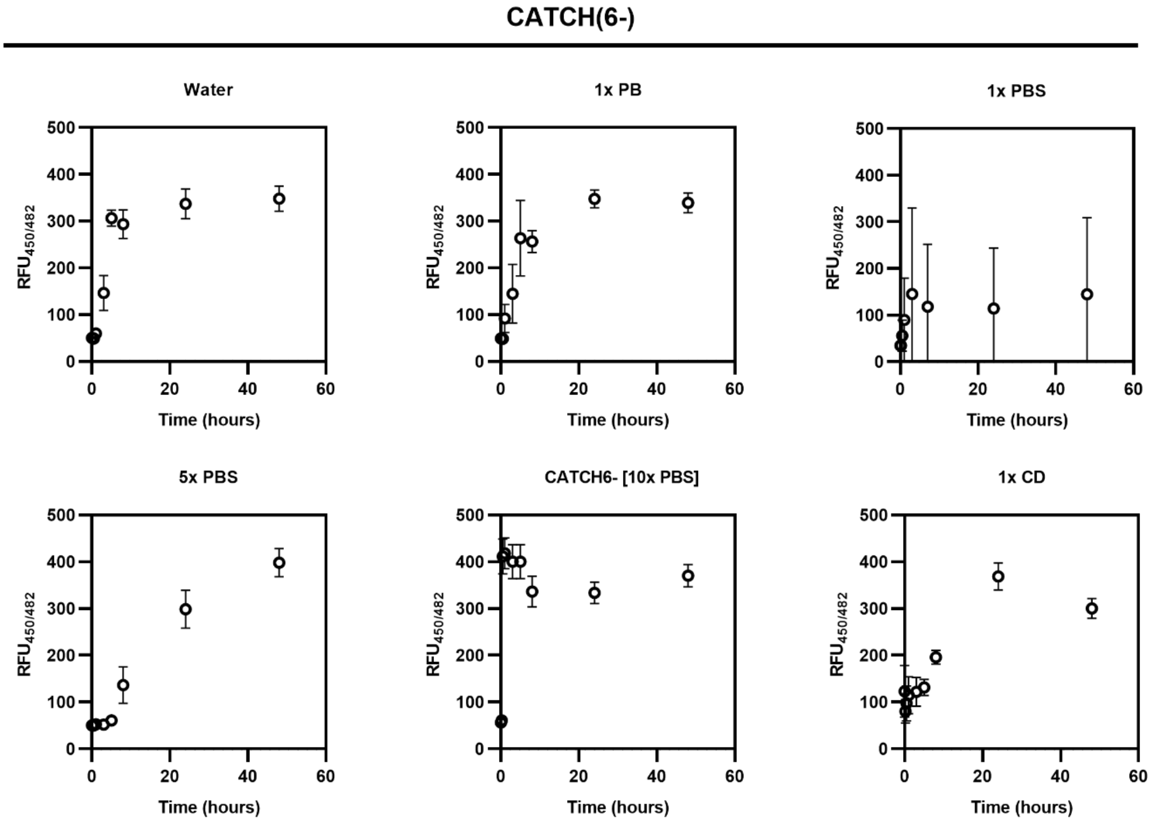

Supplementary Fig. 24: ThT kinetic profiles of CATCH(6-) in water, 1x phosphate buffer (PB) (10 mM Na_2HPO_4 and 1.8 mM KH_2PO_4, pH 7.4), 1x PBS (137 mM NaCl, 2.7 mM KCL, 10 mM Na_2HPO_4 and 1.8 mM KH_2PO_4, pH 7.4), 5x PBS, 10x PBS, and 1x CD buffer (137 mM KF, 2.7 mM KCL, 10 mM Na_2HPO_4 and 1.8 mM KH_2PO_4, pH 7.4). Error bars shown as the standard error of the mean.

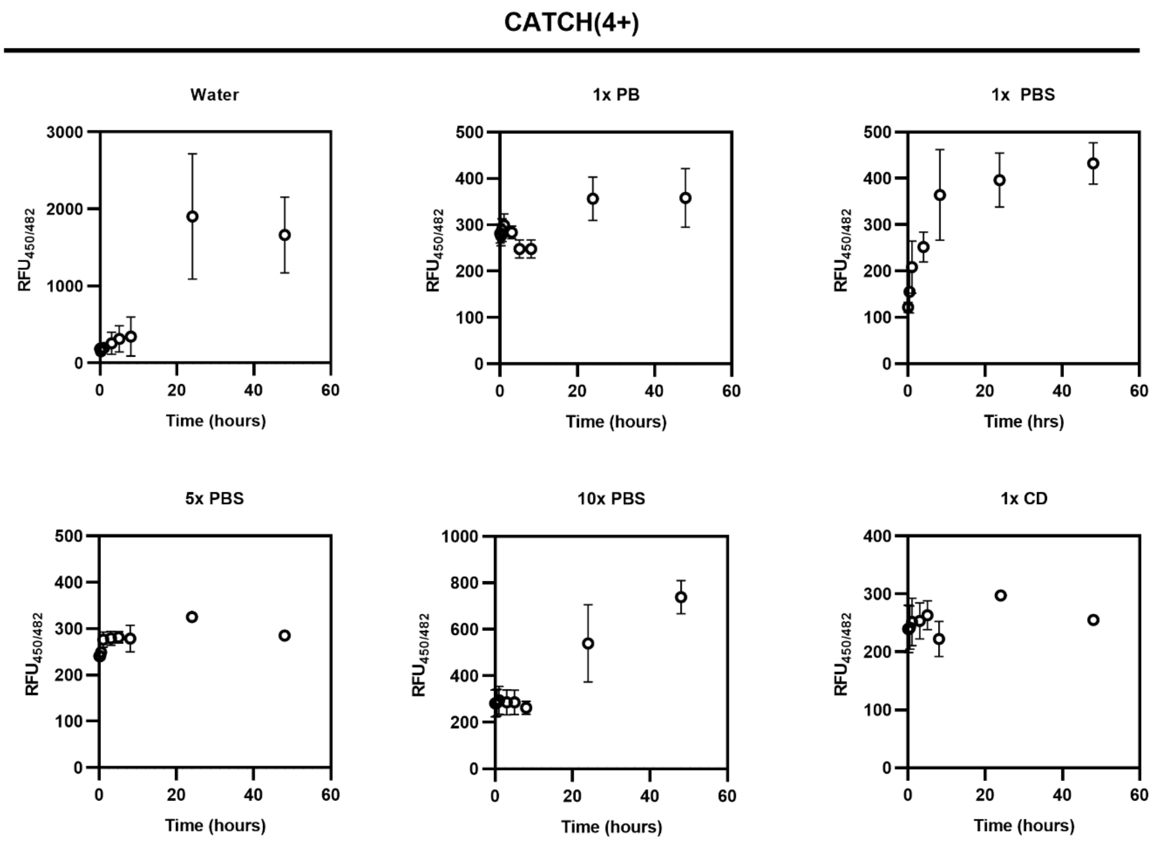

Supplementary Fig. 25: ThT kinetic profiles of CATCH(4+) in water, 1x phosphate buffer (PB) (10 mM Na_2HPO_4 and 1.8 mM KH_2PO_4, pH 7.4), 1x PBS (137 mM NaCl, 2.7 mM KCL, 10 mM Na_2HPO_4 and 1.8 mM KH_2PO_4, pH 7.4), 5x PBS, 10x PBS, and 1x CD buffer (137 mM KF, 2.7 mM KCL, 10 mM Na_2HPO_4 and 1.8 mM KH_2PO_4, pH 7.4). Error bars shown as the standard error of the mean.

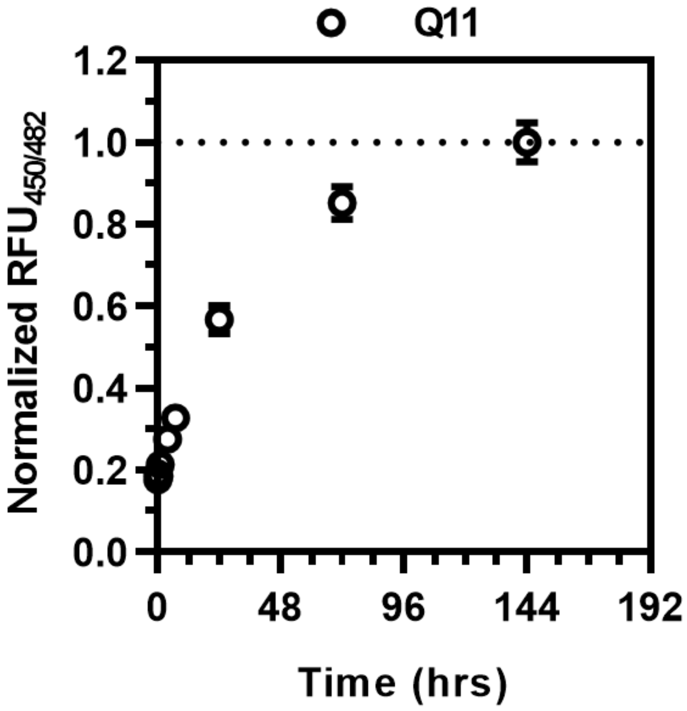

Supplementary Fig. 26: ThT kinetic assay of Q11 in 1x PBS. Error bars shown as the standard error of the mean.

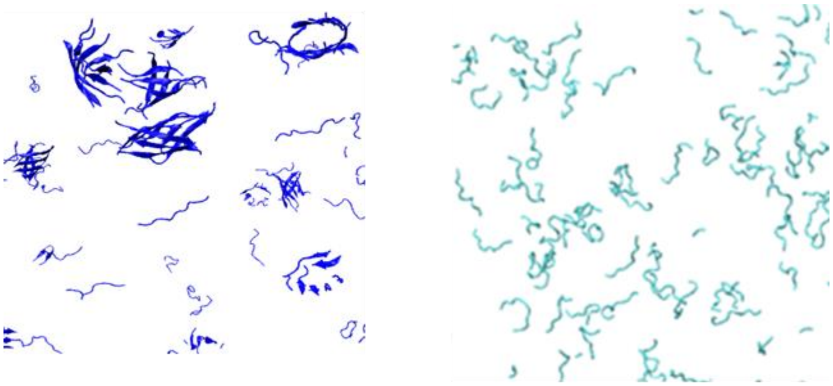

Q11 from T=0.18

Q11 from T=0.20

Supplementary Fig. 27: DMD simulation of Q11.

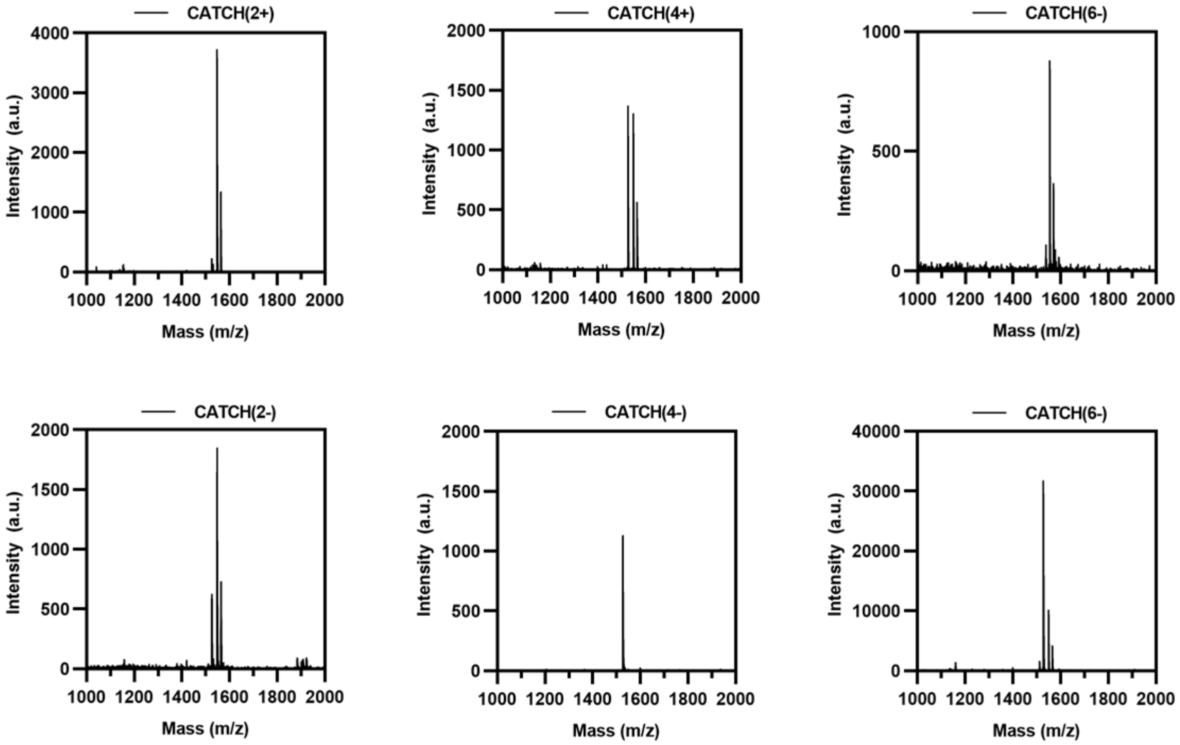

Supplementary Fig. 28: MALDI-TOF-MS spectra of peptides after RP-HPLC purification.

Supplemental Table

	CATCH(2+/2-) ^{13}C Chemical Shift (FWHM) in ppm	CATCH(4+/4-) ^{13}C Chemical Shift (FWHM) in ppm	CATCH(6+/6-) ^{13}C Chemical Shift (FWHM) in ppm
Glu C_δ Peak	179.9 (1.9)	180.9 (1.0)	180.2 (1.2)
Glu, Lys, Gln, Phe C_o Peak	171.5 (1.6)	171.5 (1.5)	171.2 (1.3)
Phe C_γ Peak	136.7 (2.3)	136.6 (2.0)	136.6 (2.3)
Glu, Lys, Gln, Phe C_α Peak	53.3 (2.9)	53.4 (2.5)	53.4 (2.7)
Lys C_δ Peak	27.8 (1.1)	27.6 (0.6)	27.7 (0.6)
Lys C_γ Peak	23.2 (1.0)	22.9 (1.1)	23.1 (1.2)

Supplemental Table 1: Analysis of select chemical shift peaks from 1D ^{13}C NMR spectra of CATCH(2+/2-), CATCH(4+/4-), and CATCH(6+/6-). Peak positions are shown along with linewidths in parentheses. All peaks are consistent within peak linewidths.
