# Supplementary material for: Charge guides pathway selection in β-sheet fibrillizing peptide co-assembly
Source: Commun Chem. 2020 Nov 13;3:172. doi: 10.1038/s42004-020-00414-w (PMC9814569; doi:10.1038/s42004-020-00414-w)
Supplement: Supplementary file 2 — Description of Additional Supplementary Files [file 42004_2020_414_MOESM2_ESM.pdf]

Description of Additional Supplementary Files

File Name: Supplementary Movie 1
Description: A video of a DMD simulation of CATCH(4+/4-) co-assembly.

File Name: Supplementary Movie 2
Description: A video of a DMD simulation of CATCH(6+/6-) co-assembly.

File Name: Supplementary Data 1
Description: Initial trajectory for CATCH(2+/2-) DMD simulation.

File Name: Supplementary Data 2
Description: Final trajectory for CATCH(2+/2-) DMD simulation.

File Name: Supplementary Data 3
Description: Initial trajectory for CATCH(4+/4-) DMD simulation.

File Name: Supplementary Data 4
Description: Final trajectory for CATCH(4+/4-) DMD simulation.

File Name: Supplementary Data 5
Description: Initial trajectory for CATCH(6+/6-) DMD simulation.

File Name: Supplementary Data 6
Description: Final trajectory for CATCH(6+/6-) DMD simulation.
